# Exercise reduces immune suppression and breast cancer progression in a preclinical model

**DOI:** 10.18632/oncotarget.27464

**Published:** 2020-01-28

**Authors:** Erik Wennerberg, Claire Lhuillier, Marissa D. Rybstein, Kyle Dannenberg, Nils-Petter Rudqvist, Graeme J. Koelwyn, Lee W. Jones, Sandra Demaria

**Affiliations:** ^1^Department of Radiation Oncology, Weill Cornell Medical College, Stich Radiation Oncology, New York, NY, USA; ^2^Memorial Sloan Kettering Cancer Center, New York, NY, USA; ^3^Sandra and Edward Meyer Cancer Center, New York, NY, USA; ^4^Department of Pathology and Laboratory Medicine, Weill Cornell Medical College, New York, NY, USA; ^5^Department of Medicine, Weill Cornell Medical College, New York, NY, USA; ^*^These authors contributed equally to this work

**Keywords:** breast cancer, exercise training, immune cells, immunotherapy, myeloid-derived suppressor cells

## Abstract

Exercise is associated with favorable changes in circulating immune cells and improved survival in early-stage breast cancer patients, but the mechansims remain to be fully elucidated. Preclinical studies indicate that physical activity started before tumor injection reduces tumor incidence and progression. Here we tested whether exercise has anti-tumor effects in mice with established 4T1 mammary carcinoma, a mouse model of triple negative breast cancer. Exercise slowed tumor progression and reduced the tumor-induced accumulation of myeloid-derived suppressor cells (MDSCs). The reduction in MDSCs was accompanied by a relative increase in natural killer and CD8 T cell activation, suggesting that exercise restores a favorable immune environment. Consistently, exercise improved responses to a combination of programmed cell death protein 1 (PD-1) blockade and focal radiotherapy. These data support further investigations of exercise in breast cancer patients treated with combinations of immunotherapy and cytotoxic agents to improve cancer outcomes.

## INTRODUCTION

Epidemiological studies suggest higher levels of exercise after a diagnosis of early breast cancer are associated with decreased risk of recurrence and cancer specific mortality [1–3]. The potential anticancer impact of exercise is supported by preclinical studies in which different exercise paradigms inhibit tumor growth across a range of genetic and transplantable tumor models, including models of breast cancer [4], although not all studies report growth inhibition. In prospective clinical trials structured exercise training induces alterations in the number and function of circulating cells of the innate immune system and, to a lesser degree, of the adaptive immune system in patients with early-stage solid tumors [5]. Whether these alterations in the systemic milieu extend to the tumor microenvironment (TME) has not been well defined. In the syngeneic B16F10 melanoma mouse model, physical activity prior to tumor implantation reduced tumor growth by enhancing mobilization and trafficking of natural killer (NK) cells [6]. In a recent study, physical activity started 8 weeks prior to tumor implantation delayed tumor growth and significantly improved survival only when used together with energy restriction in mice bearing 4T1 tumors [7], an aggressive and spontaneously metastatic model of human triple-negative breast cancer (TNBC) [8]. Whether exercise modulates the local and systemic immune environment that has been perturbed by the presence of an established tumor has not been discerned [9].

## RESULTS

**Figure 1:**
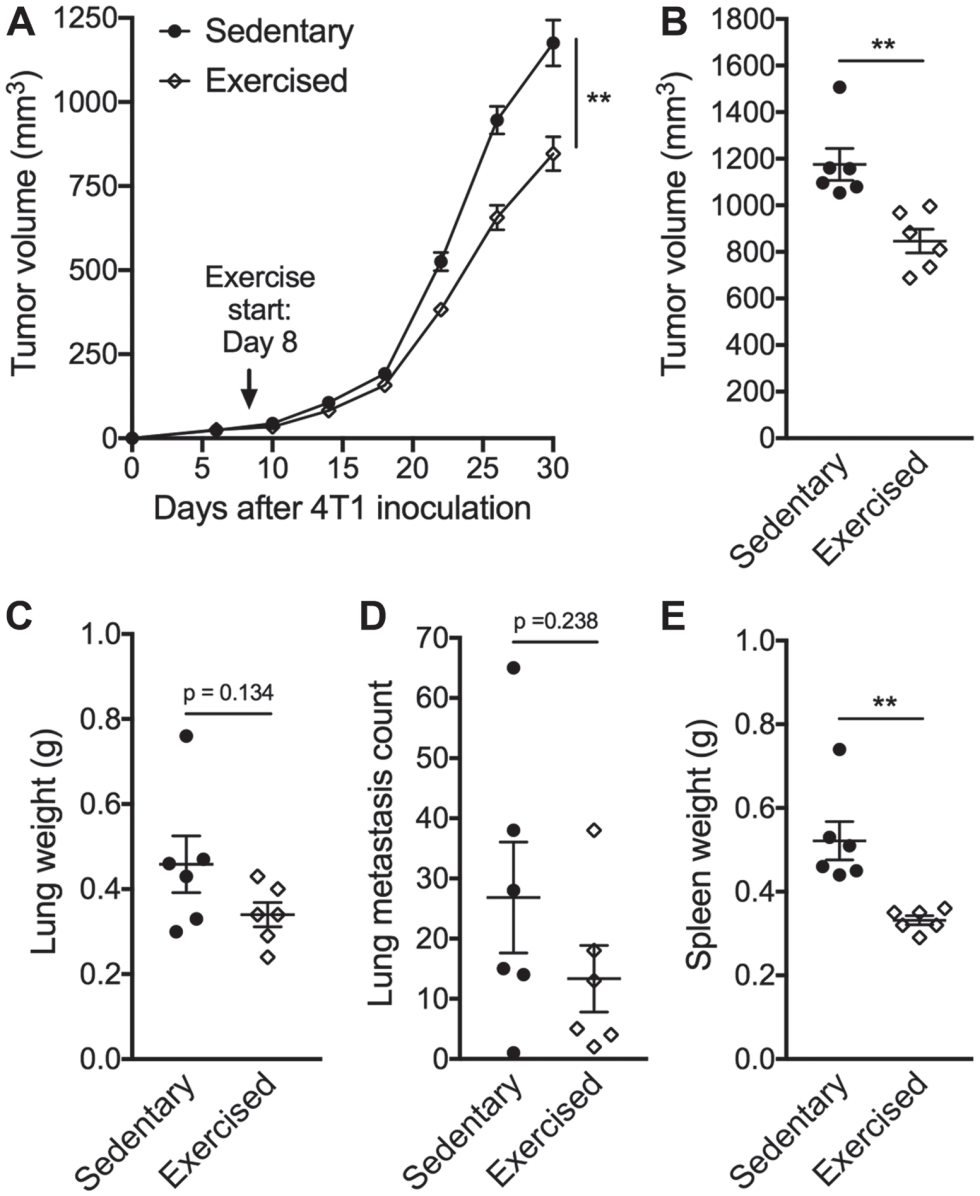
Exercise training delays tumor growth. BALB/c mice bearing subcutaneous 4T1 mammary tumors were randomly assigned into exercised or sedentary groups (*n* = 6/group). Treadmill running started on day 8 with a duration of 30 min per day (speed = 18 m/min) until the end of the experiment. (**A**) Tumor volume measurements over time. Statistically significant effect of exercise training on tumor progression was assessed by repeated measure ANOVA from start of exercise (day 8) until end of treatment (day 30). (**B**) Tumor volume, (**C**) Lung weight, (**D**) gross metastatic lung nodule count and (**E**) spleen weight measured on day 30. All data are expressed as mean ± SEM. ^**^ *p* < 0.01 with unpaired two-tailed Student’s *t*-test.

**Figure 2:**
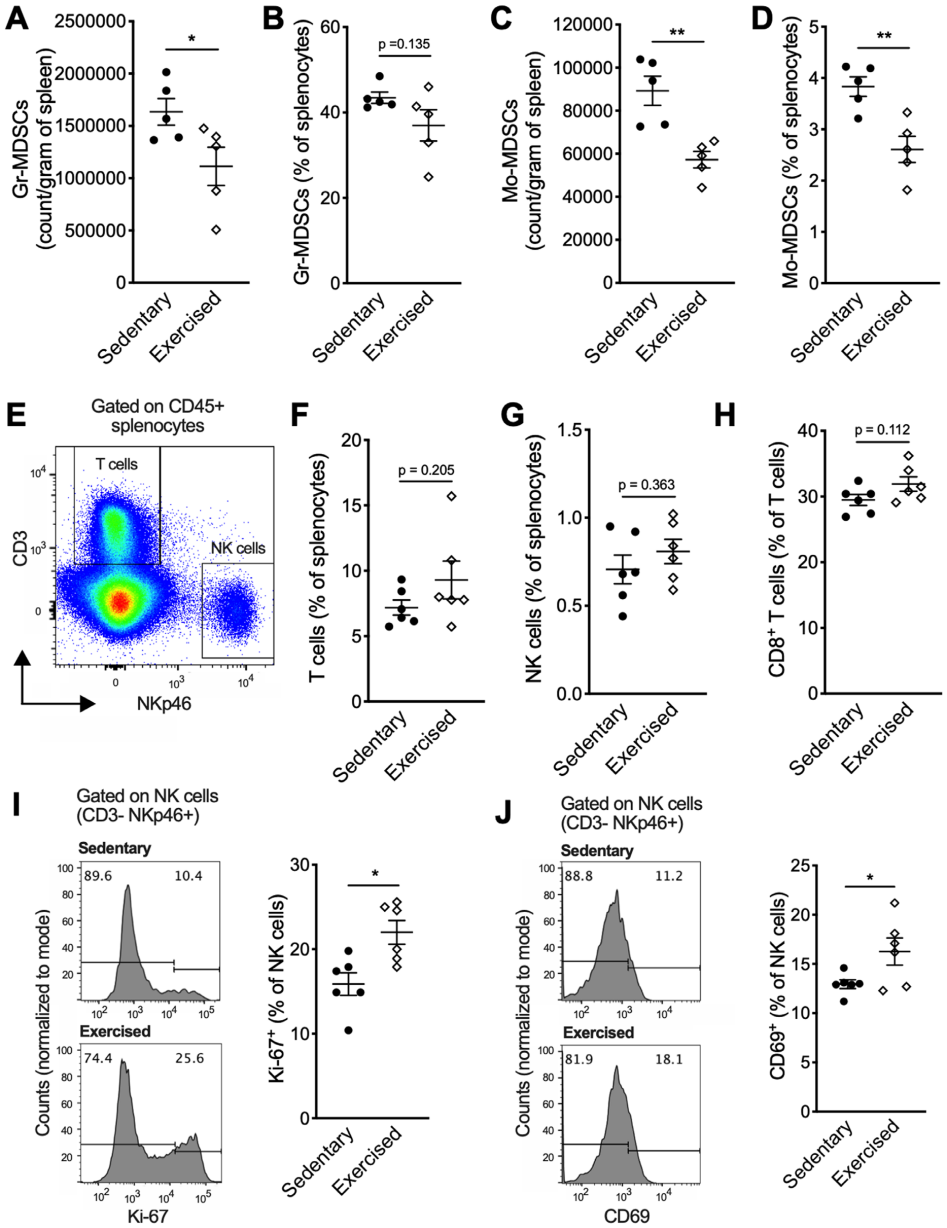
Exercise training modifies the splenic immune landscape. (**A**) Absolute Gr-MDSCs counts per gram of spleen and (**B**) frequency of Gr-MDSCs among total splenocytes on day 10 after exercise start (*n* = 5). (**C**) Absolute Mo-MDSCs counts per gram of spleen and (**D**) frequency of Mo-MDSCs among total splenocytes on day 10 after exercise start (*n* = 5). (**E**) Gating strategy to identify splenic NK cells (defined as CD3^-^ NKp46^+^) and T cells (defined as CD3^+^ NKp46^-^). (**F**) Percentages of T cells and (**G**) NK cells among total splenocytes. (**H**) Percentages of CD8^+^ T cells among splenic T cells. (**I–J**) Expression of proliferation and activation markers on splenic NK cells (gate on CD3^-^ NKp46^+^) from tumor-bearing mice on day 22 after exercise start. (I) Representative histograms of Ki-67 expression and percent Ki-67^+^ NK cells. (J) Representative histograms of CD69 expression and percent CD69^+^ NK cells. Data are expressed as mean ± SEM. ^*^ *p* < 0.05, ^**^ *p* < 0.01 with unpaired two-tailed Student’s *t*-test.

Here we tested the effects of exercise training, dosed by treadmill running at 18 m/min, 30 min per session, 5 days/week, commenced on day 8 post-implantation, when tumors became palpable. Tumor growth was significantly slower in mice treated with exercise compared to sedentary control (*p* < 0.01, Figure 1A). On day 30, tumor volume was 847 ± 124 mm^3^ in the exercise group compared to 1176 ± 168 mm^3^ in the control group (*p* < 0.01, Figure 1B). A non-signficant reduction in lung weight and number of surface metastases were also observed in the exercise group (Figure 1C–1D). 4T1 tumor progression is associated with marked splenic accumulation of myeloid-derived suppressor cells (MDSCs) and splenomegaly [10]. This phenotype was attenuated in the exercise group as characterized by smaller spleens compared to sedentary controls (0.33 ± 0.03 g vs 0.52 ± 0.11 g respectively; *p* < 0.01) (Figure 1E). Flow cytometry analysis of splenic cells showed frequencies as well as absolute numbers of both granulocytic MDSCs (Gr-MDSCs) and monocytic MDSCs (Mo-MDSCs) were significantly reduced in the exercise group (Figure 2A–2D). There were no significant differences in percentages of splenic T cell subsets and NK cells (Figure 2E–2H); however, proliferating and activated NK cells, as determined by Ki-67 and CD69 expression, respectively (Figure 2I–2J) were significantly elevated following exercise treatment.

**Figure 3:**
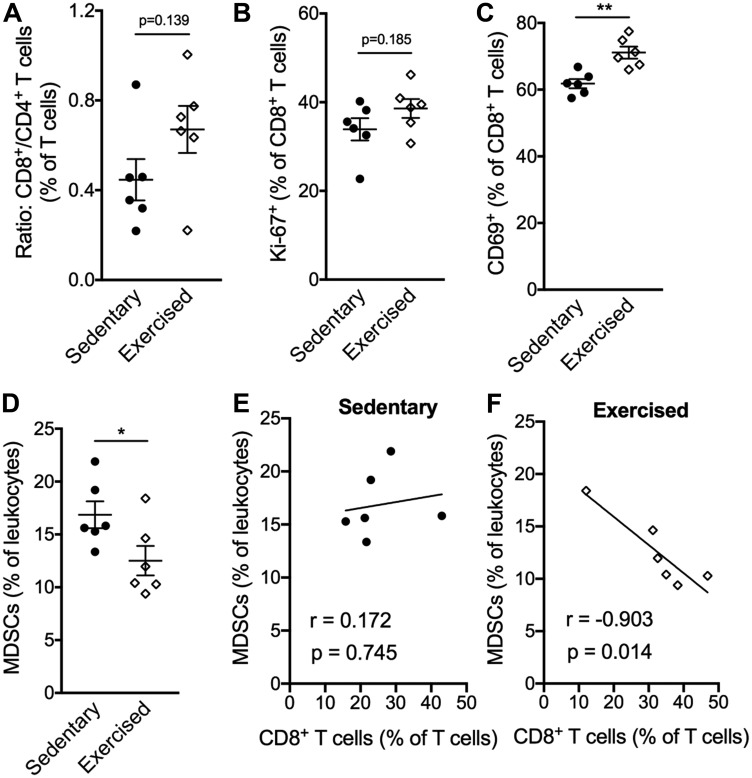
Exercise training alters the compostion of intratumoral leukocytes favoring CD8^+^ T cells. (**A–F**) Percentages and cell ratios among leukocytes isolated from tumors on day 22 after exercise start. (A) Ratio of CD8+ T cells over CD4+ T cells. (B) Percentage of Ki-67+ and (C) CD69+ cells among CD8+ T cells. (D) Percentage of total MDSCs (Gr-MDSCs and Mo-MDSCs) among total leukocytes. (E–F) Correlation analysis of MDSCs (% of leukocytes) and CD8+ T cells (% of T cells) in sedentary (E) and exercised mice (F), Pearson correlation coefficient was used to determine the association between MDSCs and CD8+ T cell ratios. All data are expressed as mean ± SEM. *p < 0.05; **p < 0.01 with unpaired two-tailed Student’s *t*-test.

We next analyzed whether reduced systemic immune suppression occurred in conjunction with changes in the TME. Exericse treatment increased the CD8/CD4 T cell ratio compared to sedentary mice (Figure 3A) although this was not significant. CD8^+^ T cells did not show differences in proliferation by Ki-67 staining, but they were more activated as shown by expression of CD69 in tumors following exercise treatment (Figure 3B–3C). A reduced frequency of MDSCs was seen in tumors of mice treated with exercise (Figure 3D). Finally, an inverse correlation between MDSCs and CD8^+^ T cells was observed following exercise treatment (r = 0.903; *p* = 0.014; Figure 3F) but not control (Figure 3E), suggesting that exercise shifts the balance between immune suppressive and anti-tumor effector cells in the TME.

**Figure 4:**
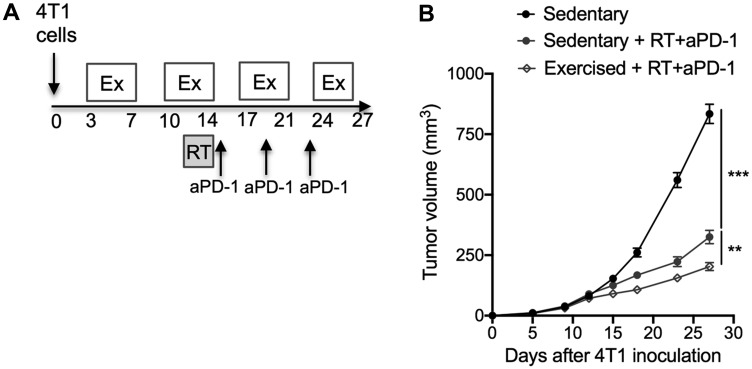
Exercise training as an adjunct therapy potentiates anti-tumor immunity induced by radiotherapy and immunotherapy combinations. (**A**) Experimental procedure: mice were randomly assigned to the following groups: sedentary, sedentary + RT/aPD-1 and exercised + RT/aPD-1 (*n* = 10/group). 4T1 tumor cells (5 × 10^4^) were injected subcutaneously in the right flank of mice on day 0. Tumors were irradiated in three doses of 8 Gy each on 3 consecutive days. Anti–PD-1 antibodies were given intraperitoneally at 200 μg/mouse on days 15, 19 and 23 post tumor implantation. (**B**) Tumor growth over time. Statistically significant effect of exercise training on tumor progression was assessed by repeated measure ANOVA from start of exercise (day 3) until end of treatment (day 27). All data are expressed as mean ± SEM. ^**^ *p* < 0.01, ^***^ *p* < 0.001.

**Figure 5:**
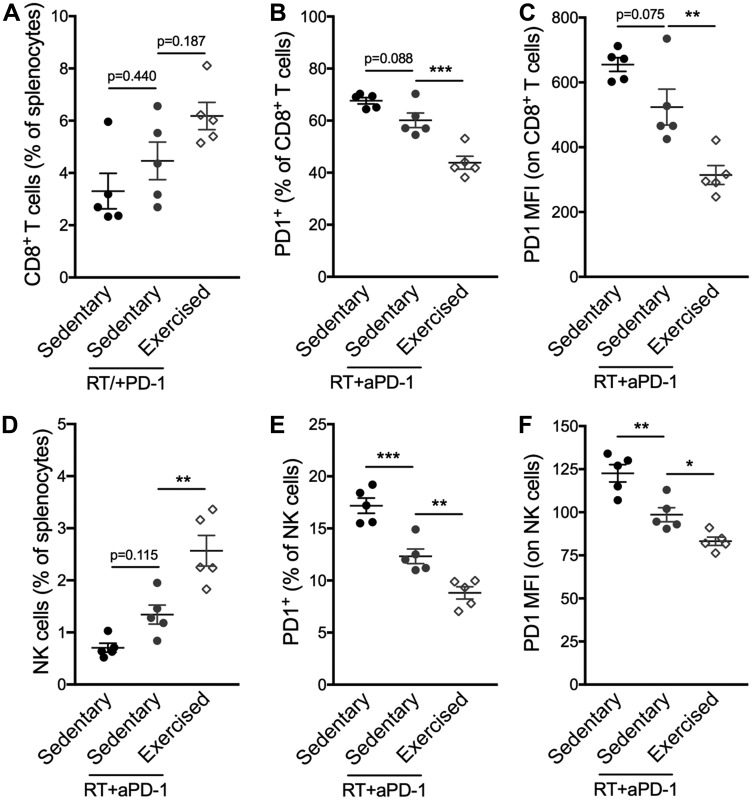
Exercise training following RT/aPD-1 therapy promotes splenic infiltration and reduced PD-1 expression of CD8^+^ T cells and NK cells. (**A–F**) Percentages and expression levels assessed by mean fluorescence intensity (MFI) on CD8^+^ T cells and NK cells from spleens harvested from tumor-bearing mice on day 27. (A) Percentage of CD8^+^ T cells among splenocytes. (B) Percentage of PD-1^+^ CD8^+^ T cells and (C) PD-1 MFI on CD8^+^ T cells. (D) Percentage of NK cells among splenocytes. (E) Percentage of PD-1^+^ NK cells and (F) PD-1 MFI on NK cells. All data are expressed as mean ± SEM. ^*^ *p* < 0.05; ^**^ *p* < 0.01, ^***^ *p* < 0.001 with ordinary one-way ANOVA and Tukey’s multiple comparisons test.

**Figure 6:**
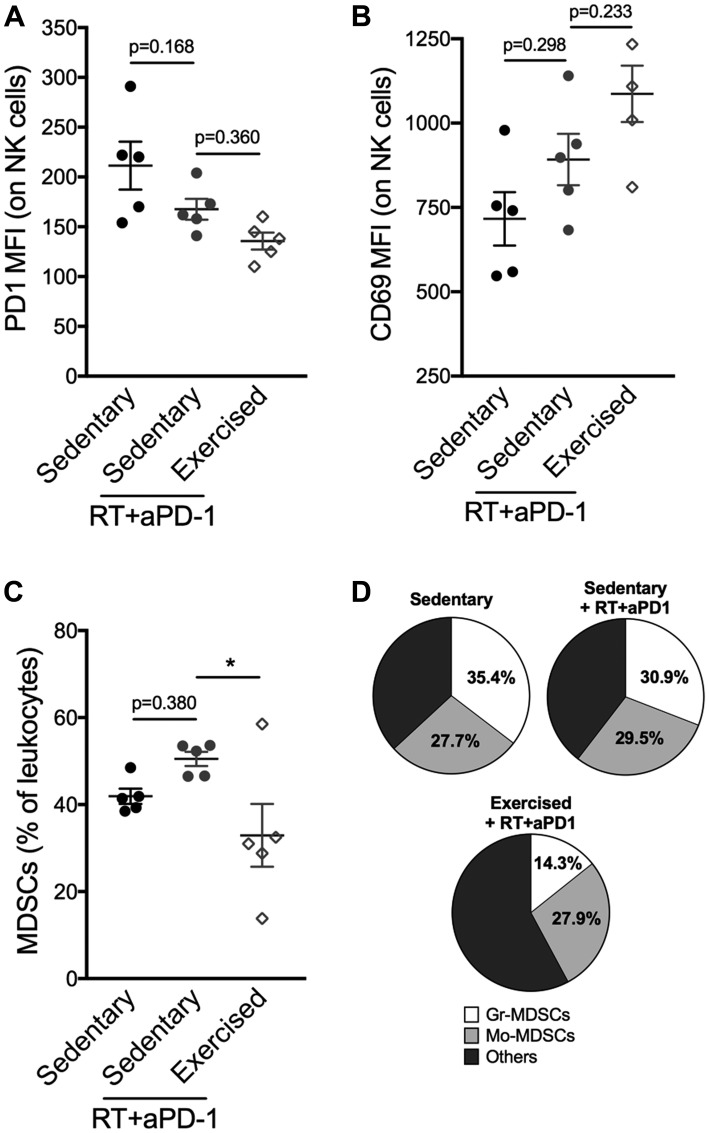
Exercise training following RT/aPD-1 therapy reduces intratumoral MDSCs accumulation and favors NK cell activation. (**A–B**) Flow cytometry analysis of tumor-infiltrating NK cells on day 27: (A) PD-1 and (B) CD69 expression assessed by mean fluorescence intensity (MFI). (**C**) Percentages of total MDSCs (Gr-MDSCs and Mo-MDSCs) in tumors and (**D**) specific proportions of Gr-MDSCs and Mo-MDSCs among myeloid cells (CD11b^+^). ^*^ *p* < 0.05 with ordinary one-way ANOVA and Tukey’s multiple comparisons test.

Given the exercise-induced changes in immune compostition in favor of lymphocyte activation at the level of the whole organism as well as the TME, we hypothesized that response to immunotherapy would be enhanced. Similarly to the majority of TNBC patients, 4T1 tumors fail to respond to programmed cell death protein 1 (PD-1) blockade alone [11, 12]. However, PD-1 blockade enhances the response of the tumor to focal radiotherapy (RT) [12]. Thus, we tested the effect of exercise in combination with RT and PD-1 blockade. The triple combination treatment of exercise+RT+PD-1 blockade was associated with significantly slower tumor growth compared to the dual combination of RT+PD-1 blockade (Exercise+RT+aPD-1: 203 ± 52 mm^3^ vs sedentary+RT+aPD-1: 325 ± 83 mm^3^; *p* < 0.01 Figure 4A–4B). CD8^+^ T cells were increased in the spleen in exercised compared to sedentary mice treated with RT+aPD-1 (Figure 5A), and expressed significantly less PD-1 (Figure 5B–5C). Likewise, spleen NK cells were increased in the triple combination versus dual combination group (Figure 5D). In addition, PD1 expression was lowest on NK cells from the triple combination group in both the spleen and TME (Figure 5E–5F and Figure 6A). NK cells present in the TME from exercised mice treated with RT+aPD-1 also showed the highest expression of CD69, suggestive of a more activated state (Figure 6B). Importantly, exercise significantly reduced MDSCs in RT+aPD-1-treated mice, largely due to a marked reduction in Gr-MDSCs (Figure 6C–6D).

## DISCUSSION

A few pre-clinical studies have shown that physical activity modulates the immune contexture and delays tumor growth when commenced prior to tumor inoculation [6, 13, 14]. We have previously shown that “prescribed” exercise started at the time of tumor inoculation delayed tumor growth in mouse breast cancer models [15]. Here we demonstrated that exercise started when the tumor is established improves tumor control and restores at least in part the immune balance that is compromised in tumor-bearing hosts.

Increased NK cell activation was reported in exercised mice and patients [6, 16]. NK cells control micrometastatic disease [17, 18] and one can speculate that they may have contributed to the observed trend towards reduced metastatic burden in exercised mice. The most notable effect of exercise training was a decrease in MDSCs in both the spleen and the tumor. A reduction in MDSCs was recently reported in 4T1 tumor-bearing mice that was proportional to the level of physical activity and observed only in combination with energy restriction [7]. Thus, exercise training may be more effective than voluntary physical activity at reducing the immune suppression associated with tumor progression, but we cannot exclude that effectiveness may depend on the exercise dose. We used an exercise regimen associated with physiological adaptation in skeletal muscle, which approximately equates to a moderate-intensity exercise for mice, based on prior work [19, 20]. Importantly, we found that exercise enhanced tumor response to focal RT and PD-1 blockade, suggesting that it could be a component of a multi-modality treatment for breast cancer that includes immunotherapy. The effect of exercise on tumor response to RT or PD-1 blockade used alone was not tested here, and this important question will need to be addressed using breast cancer models with different degree of intrinsic radiosensitivity and immunogenicity.

The aggressive behavior of TNBC has been associated with increased MDSCs in mice and patients [21], suggesting that the benefits of exercise mediated via the reduction in MDSCs that we have shown here may be relevant to the clinic. However, we recognize that our study is limited by the use of a single tumor model, and exercise has been shown to differentially affect tumor growth in different mouse models, likely due to inherent differences in tumor metabolism [19]. Interestingly, a link between breast cancer cell metabolism and induction of MDSCs has been recently described [22], warranting further investigations into the ability of exercise to subvert TNBC metabolism.

In sum, we demonstrate that exercise training in a preclinical model of established TNBC induces favorable immunological changes, systemically and in the TME, delays tumor progression, and improves the response to a combination of radiotherapy and PD-1 blockade. These results have potential implications for the treatment of patients with atezolimumab which was recently FDA approved in combination with chemotherapy for patients with TNBC [23]. Further investigations on the value of exercise training in breast cancer patients are warranted.

## MATERIALS AND METHODS

### Cells and reagents

BALB/c mouse-derived poorly immunogenic mammary carcinoma 4T1 cells were obtained from F. Miller [8]. Cells were authenticated by morphology, growth, and pattern of metastasis *in vivo* and routinely screened for Mycoplasma (LookOut Mycoplasma PCR Detection kit, Sigma-Aldrich). 4T1 cells were cultured in DMEM (Life Technologies) supplemented with L-glutamine (2 mmol/L), penicillin (100 U/mL), streptomycin (100 μg/mL), 2-mercapthoethanol (2.5 × 10^−5^ mol/L), and 10% FBS (Life technologies). Anti-mouse PD-1 (clone RMP1-14, Cat # BE0146) monoclonal antibody (mAb) was purchased from BioXCell.

### Mouse model and exercise interventions

Six- to eight-week-old female BALB/c mice were obtained from Taconic (Germantown, NY, USA). All experiments were approved by the Institutional Animal Care and Use Committee. Mice were subcutaneously (s. c.) inoculated with 4T1 cells (5 × 10^4^) and assigned to treatment groups (*n* = 6/group): exercised and sedentary. Eight days after tumor inoculation, mice were exercised on a treadmill five days a week until the end of the experiment. For each session of running, the treadmill speed was set to 18 m/min for a total of 30min. Mice were exercised at the same time each day. Sedentary mice were kept in their home cages during exercise. All mice were provided food and water ad libitum. Perpendicular tumor diameters were measured with a Vernier caliper and tumor volumes were calculated as length×width^2^×0.52. Mice were sacrificed 30 days after tumor inoculation for immune phenotyping of the tumors and the spleens. Lungs were also harvested and fixed in 4% paraformaldehyde before counting of metastases.

### Exercise in combination with RT and PD-1 blockade

Mice were randomly assigned to one of the three groups (*n* = 10/group): no exercise/no treatment, RT and PD-1 blockade (aPD-1), or exercise plus RT and aPD-1. Tumors were irradiated as previously described using the Small Animal Radiation Research Platform (SARRP Xstrahl Ltd) in three doses of 8 Gy each on 3 consecutive days [12]. Anti–PD-1 mAb was given intraperitoneally (i.p.) at 200 μg/mouse on days 15, 19 and 23 post tumor inoculation.

### Flow cytometry analysis

Tumors were enzymatically digested using a Mouse Tumor Dissociation Kit (Miltenyi Biotec) on a gentleMACS Octo Dissociator (Miltenyi Biotec). Digested tumors and single-cell suspensions from spleens were filtered through a 40 μm strainer to remove large debris. Red blood cell lysis was then performed on splenic suspensions (Invitrogen). Cells were blocked with CD16/32 antibody to Fc gamma III and II receptors (Thermo Fisher Scientific, Clone 93) and were then stained for surface markers using the following fluorochrome-conjugated antibodies and dyes; Fixable viability dye-eFluor 780, CD3-eFluor 450, PD-1-FITC, CD69-PerCP-Cy5.5, NKp46-APC (eBioscience), CD45-V500 (BD Biosciences), CD8b-PE-Vio770, Ly6G-APC-vio770, CD11b-PE-vio770, Ly6C-FITC (Miltenyi Biotec). Counting beads (Countbright, Invitrogen) was added to the samples prior to acquisition. After surface marker staining, cells were fixed using IC fixation buffer (Invitrogen) and permeabilized using a permabilization buffer (Invitrogen) followed by staining for intracellular targets (Ki-67-PE). Samples were acquired with a MACSQuant^®^ Analyzer 10 (Miltenyi Biotec), and flow data was analyzed using FlowJo software (version 8.8.7). When determining mean fluorescence intensity (MFI) for activation and proliferation markers, elimination of potential differences in autofluorescence between treatment groups and individual samples was assured by subtracting the MFI of stained cells with the MFI of cells stained with fluorescence minus one (FMO) for each sample.

### Statistics

Data are presented as mean ± standard error of the mean (s. e. m.). For comparisons between two groups, *p* values were calculated using unpaired two-sided Student’s *t*-test. Pearson correlation analysis was used to determine the association between cell ratios. Differences in tumor volume between treatment groups was evaluated using repeated measures Analysis of Variance (ANOVA) from exercise start until mice were sacrificed. Differences were considered significant at *p* values below 0.05. All data were analyzed using GraphPad Prism software (GraphPad version 7).
